# Quantitative Biodistribution and Pharmacokinetics Study of GMP-Grade Exosomes Labeled with ^89^Zr Radioisotope in Mice and Rats

**DOI:** 10.3390/pharmaceutics14061118

**Published:** 2022-05-24

**Authors:** Hojun Choi, Myung-Yoon Kim, Dae-Hwan Kim, Hanoul Yun, Byung-Koo Oh, Su-Bin Kim, In-Ho Song, Hyun-Soo Park, Sang-Eun Kim, Cheolhyoung Park, Chulhee Choi

**Affiliations:** 1 ILIAS Biologics Inc., Daejeon 34014, Korea; hchoi@iliasbio.com (H.C.); mykim@iliasbio.com (M.-Y.K.); dkim@iliasbio.com (D.-H.K.); hyun@iliasbio.com (H.Y.); bkoh@iliasbio.com (B.-K.O.); 2 Department of Applied Bioengineering, Graduate School of Convergence Science and Technology, Seoul National University, 1 Gwanak-ro, Gwanak-gu, Seoul 08826, Korea; ssuvin721@snu.ac.kr; 3 Department of Nuclear Medicine, Seoul National University Bundang Hospital 82, Gumi-ro 173 Beon-gil, Bundang-gu, Seongnam 13620, Korea; 99269@snubh.org (I.-H.S.); hyuns@snu.ac.kr (H.-S.P.); kse@snu.ac.kr (S.-E.K.); 4 Department of Molecular Medicine and Biopharmaceutical Sciences, Graduate School of Convergence Science and Technology, Seoul National University, 1 Gwanak-ro, Gwanak-gu, Seoul 08826, Korea; 5 Advanced Institutes of Convergence Technology 145, Gwanggyo-ro, Yeongtong-gu, Suwon 16229, Korea; 6 Department of Bio and Brain Engineering, Korea Advanced Institute of Science and Technology (KAIST), Daejeon 34141, Korea

**Keywords:** exosome, biodistribution, pharmacokinetics, zirconium, positron emission tomography/computed tomography

## Abstract

For the successful clinical advancement of exosome therapeutics, the biodistribution and pharmacokinetic profile of exogenous exosomes in various animal models must be determined. Compared with fluorescence or bioluminescence imaging, radionuclide imaging confers multiple advantages for the in vivo tracking of biomolecular therapeutics because of its excellent sensitivity for deep tissue imaging and potential for quantitative measurement. Herein, we assessed the quantitative biodistribution and pharmacokinetics of good manufacturing practice-grade therapeutic exosomes labeled with zirconium-89 (^89^Zr) after systemic intravenous administration in mice and rats. Quantitative biodistribution analysis by positron emission tomography/computed tomography and gamma counting in mice and rats revealed that the total ^89^Zr signals in the organs were lower in rats than in mice, suggesting a higher excretion rate of exosomes in rats. A prolonged ^89^Zr signal for up to 7 days in most organs indicated that substantial amounts of exosomes were taken up by the parenchymal cells in those organs, highlighting the therapeutic potential of exosomes for the intracellular delivery of therapeutics. Exosomes were mainly distributed in the liver and to a lesser extent in the spleen, while a moderately distributed in the kidney, lung, stomach, intestine, urinary bladder, brain, and heart. Exosomes were rapidly cleared from the blood circulation, with a rate greater than that of free ^89^Zr, indicating that exosomes might be rapidly taken up by cells and tissues.

## 1. Introduction

In recent decades, the field of nanomedicine has experienced rapid advances in therapeutic applications for various diseases [1]. Extracellular vesicles (EVs) have attracted increasing attention as novel intracellular delivery vehicles for various bioactive molecules [2,3]. EVs are cell-derived lipid nanoparticles that can be categorized into exosomes, microvesicles, and apoptotic bodies based on their biological properties and biogenesis pathways [4,5,6]. However, careful interpretation is warranted when distinguishing between EV subgroups because most EV isolation methods are based on biological properties, such as size and density, and not on their unique biogenesis pathways [6]. Thus, the EV research society recommends clearly defining the subtypes of EVs used in each study [6]. Exosomes, or small EVs, are membranous vesicles composed of a single phospholipid bilayer formed by inward invagination from endosomes and typically range from 50 to 150 nm [7]. Exosomes are present in almost all body fluids and actively participate in cell-to-cell communication by transporting diverse bioactive molecules, such as nucleic acids (DNA and RNA), proteins, and lipids [8,9,10,11,12,13]. Among the subtypes of EVs, exosomes have attracted increasing attention in terms of their therapeutic potential because of their role in delivering diverse biological molecules for intercellular communication. Exosomes have advantages as therapeutics in terms of biocompatibility, low immunogenicity, and efficient intracellular delivery [14,15]. As exosomes inherit many physiological properties of its parental cells, exosomes can be used as cell-free therapeutics with improved delivery and safety profiles compared to cell therapy [16,17,18,19]. Numerous efforts have been made to use exosomes as drug delivery vehicle by engineering exosomes or exosome-producing cells to efficiently incorporate active pharmaceutical ingredients [15,20,21]. Approximately 50 clinical trials have assessed the safety and efficacy of either naïve or engineered exosomes in different therapeutic areas. 

To develop new therapeutic modalities, determining the biodistribution and pharmacokinetic profiles in various animal models is crucial for successful clinical translation. Systemically administered exosomes have been reported to be mainly distributed to the liver and spleen and to a lesser extent to the kidney, lung, and gastrointestinal tract; however, the distribution can be altered by various factors, such as the cellular origin of the exosomes and composition of the exosomal membranes (e.g., surface proteins, lipids, and glycans) [22,23,24,25,26,27,28,29,30]. In addition, the surface of exosomes can be further engineered to induce targeted delivery to desired cells or organs, including the brain, placenta, heart, spinal cord, and cartilage [31,32,33,34,35,36]. Prolonged retention of exosomes is observed in tissues, such as of the liver and spleen, which show sustained retention for longer than 24 h [22,24,27]. Nonetheless, careful interpretation is needed to analyze the tissue pharmacokinetics (PK) of exosomes because most exosome imaging techniques utilize methods in which the lipid bilayer of exosomes is labeled with various imaging dyes, which may lead to tracking of the cell-ingested phospholipids and not the exosome itself. Systematically administered exosomes demonstrate rapid clearance from the blood circulation, with a half-life of less than a few minutes in healthy animals, which is primarily due to rapid clearance by cells of the mononuclear phagocyte system, such as macrophages and neutrophils [24,27,37]. 

Currently, fluorescence or luminescence imaging is widely used to monitor the in vivo biodistribution of administered exosomes. Exosomes can be labeled with various lipophilic fluorescent dyes, such as DiR and DiD, to track exosomes in vivo [22,38,39]. However, fluorescence- or luminescence-based exosome tracking suffers from limitations regarding poor tissue penetration and difficulties in quantification due to signal reduction over time [40]. With recent technological advancements in deep tissue penetration imaging, other imaging methods, including magnetic resonance imaging (MRI), positron emission tomography (PET), and single photon emission computed tomography (SPECT), are also being utilized to monitor the biodistribution and PK of exosomes [22,41,42,43,44,45]. Radionuclide imaging confers multiple advantages over fluorescence or bioluminescence imaging for in vivo tracking of radiolabeled therapeutics because of its excellent sensitivity for deep tissue imaging and its potential for quantitative measurement. A study comparing fluorescent, bioluminescent, and radioactive tracers for tracking EVs administered in vivo revealed that radiolabeling is the most accurate EV tracking approach for quantitative biodistribution studies [46]. Most studies that use radionuclide imaging to analyze exosome biodistribution, label radioisotopes on the surface of the exosomes. Morishita et al. analyzed the quantitative biodistribution of B16BL6-derived exosomes in mice by radiolabeling the surface of exosomes with iodine-125 (^125^I) based on a streptavidin-biotin system, and they measured the time-dependent organ distribution using a gamma counter [47]. González et al. radiolabeled the surface of milk-derived exosomes using technetium-99m (^99m^Tc) for in vivo tracking in mice [48], and Jung et al. also labeled the surface of mouse breast cancer-derived exosomes with ^64^Cu (or ^68^Ga) and visualized in vivo-administered exosomes by PET imaging in mice [49]. While most studies radiolabel the surface of exosomes for in vivo tracking, some studies use the intraluminal labeling method. Hwang et al. radiolabeled ^99m^Tc into the lumen of macrophage-derived exosome-mimetic nanovesicles, which are cell-derived nanoparticles generated by the extrusion of cells, for an in vivo biodistribution analysis in mice [50]. Khan et al. intraluminally labeled zirconium-89 (^89^Zr) by complexing ^89^Zr with 8-hydroxyquinoline (8-HQ), which allowed for the delivery of ^89^Zr across the lipid bilayer of exosomes [51]. Faraqu et al. revealed that labeling exosome membranes with ^111^indium showed higher efficiency and radiochemical stability than the intraluminal labeling method [27]. Careful consideration of the stability of radiolabeling is required for biodistribution studies because the observer might track free radioisotopes that have been detached from exosomes.

In this study, we assessed the quantitative biodistribution and PK of good manufacturing practice (GMP)-grade therapeutic exosomes in various tissues of mice and rats by labeling exosomes with ^89^Zr for preclinical evaluation. The characteristics of exosomes are greatly affected by the parental cell conditions and exosome purification methods [52,53,54]. In addition, rigorous exosome quality control is required to minimize batch-to-batch variance owing to EV heterogeneity. We optimized the exosome purification and quality control processes to generate GMP-grade therapeutic exosomes with minimal batch-to-batch variances. The therapeutic exosomes used in this study, ILB-202, are loaded with the super-repressor IκB (srIκB), which is an anti-inflammatory protein that inhibits the function of NF-κB [55,56]. srIκB was incorporated into exosomes via a protein loading technology called EXPLOR, which we previously developed [57]. ^89^Zr is a nearly ideal radioisotope for PET imaging because it possesses a physical half-life (T_1/2, physical_ = 78.4 h) that is compatible with the in vivo PK of exosomes [58]. Numerous successful examples of imaging studies with ^89^Zr-labeled bioactive molecules show the general applicability of this radiometal nuclide for long-term in vivo PET imaging [59,60]. Furthermore, the surface of exosomes can be readily labeled with ^89^Zr using the siderophore-derived chelator desferrioxamine (DFO) [61]. We assessed the preclinical biodistribution and PK of GMP-grade therapeutic exosomes using ^89^Zr-labeled ILB-202 (^89^Zr-Exo) radiolabeled on the surface of exosomes using DFO. We performed in vivo PET/CT imaging and ex vivo gamma-counting analyses of various organs, urine, and blood of mice and rats for 7 days after ^89^Zr-Exo administration for the quantitative analysis of exosome biodistribution.

## 2. Materials and Methods

### 2.1. Production and Isolation of Exosomes

ILB-202-producing Expi293F cells, which expresses two recombinant proteins, srIκB-CRY2 and CIBN-CD9, were cultured in a wave system for 4 days at 37 °C. The cells were exposed to blue light illumination from a 460-nm light emitting diode in a CO_2_ incubator. The harvested medium was collected and centrifuged at 2000× *g* for 10 min at 4 °C to remove the cells and cell debris, and a 0.22 µm polyether sulfone filter was used to remove the large particles. The separation of exosomes from biological culture fluids was performed via ultrafiltration and diafiltration (UF/DF). Briefly, exosomes were isolated using a combination of anion exchange and size-exclusion chromatographies. After the UF/DF process, the final exosome isolation was completed by filtering the purified product with a 0.22 µm filter.

### 2.2. Radiolabeling Exosomes with ^89^Zr

Surface labeling of exosomes was conducted by first labeling the exosomes with DFO and then labeling ^89^Zr to the exosomal surface by conjugation with the surface-bound DFO. In detail, amine-reactive DFO (p-NCS-Bn-DFO, Future Chem, Seoul, Korea) dissolved in DMSO at a concentration of 5–20 mg/mL was mixed with exosomes at a weight ratio of 1:10 (weight of DFO:weight of proteins in exosomes). The mixture was incubated for 60 min at 37 °C with shaking at 550 rpm using a thermomixer. After incubation, exosomes labeled with DFO (DFO-Exo) were purified using a dextran desalting column (molecular weight cut-off (MWCO) 5000, Pierce, WI, USA), with 10 mM phosphate-buffered saline (PBS, pH 7.4, without calcium and magnesium) as the eluent. The pH of ^89^Zr^4+^ in 1.0 M oxalic acid was adjusted to 6.8–7.5 using 1.0 M Na_2_CO_3_ and then added to DFO-Exo. The mixture was then incubated for 60 min at room temperature (550 rpm) using a thermomixer. ^89^Zr-Exo was purified using a dextran desalting column (MWCO 50,000), with 10 mM PBS (pH 7.4, without calcium and magnesium) as the eluent.

### 2.3. Determining the Radiolabeling Efficiency Using Thin-Layer Chromatography (TLC)

The radiochemical yield was calculated by dividing the amount of activity isolated from purified ^89^Zr-Exo by the amount of activity initially added to the solution. Purified ^89^Zr-Exo was dropped onto a silica-impregnated TLC strip (iTLC-SG). After the aliquot dried, TLC with 50 mM DPTA (pH 7) solution was used as the mobile phase, and the TLC strip was analyzed as the stationary phase using a radio-TLC scanner (AR-2000, Eckert & Ziegler, Berlin, Germany). ^89^Zr^4+^ bound to the DFO-Exo construct appeared at the origin (retention factor, Rf = 0.1), whereas free ^89^Zr^4+^ cations were chelated by DTPA and eluted with the solvent front (Rf = 0.9–1.0). The radiochemical purity of ^89^Zr-Exo was verified by integrating the radiochromatogram, dividing the area under the curve from Rf 0.0–0.1 by the total area under the curve, and multiplying by 100.

### 2.4. Measurement of the Size Distribution and Zeta Potential of Exosomes

Size distribution of exosomes were measured by nanoparticle tracking analysis (NTA) using NanoSight 300 (Malvern Panalytical, Malvern, UK). The samples were diluted by 1:2000 with 10 mM PBS (pH 7.4, without calcium and magnesium) for NTA analysis. The following settings were used for data acquisition: camera level 14, and detection threshold 5. Data analysis was performed by using NTA 3.4 Build 3.4.4 Software. The zeta-potential measurement was performed using Zetasizer Nano ZS (Malvern Panalytical, Malvern, UK). The samples were diluted by 1:2000 in 10 mM PBS (pH 7.4, without calcium and magnesium) and loaded into Malvern Folded Capillary Zeta Cell. All measurements were performed at 25 °C and data were analyzed using Zetasizer Software (V7.12, Malvern Panalytical Ltd., Malvern, UK).

### 2.5. Cell Culture

THP-1 cells were grown in RPMI 1640 medium containing 2-mercaptoethanol (final concentration of 0.05 mM), and HEK293 cells were maintained in Dulbecco’s modified Eagle medium. All media were supplemented with 10% fetal bovine serum and 1% antibiotic/antimycotic. Cultures were maintained at 37 °C in a 5% carbon dioxide incubator.

### 2.6. Cellular Uptake Assay of ^89^Zr-Exo

Cellular uptake studies of ^89^Zr-Exo were performed using THP-1 and HEP293 cells. THP-1 cells were seeded in a 24-well plate (5 × 10^5^ cells/well, in triplicate), and HEK293 cells were seeded in a 6-well plate (6 × 10^5^ cells/well, in triplicate). One microgram of ^89^Zr-Exo was then added to each well. Neutralized [^89^Zr]Zr (oxalate)4 (with equal radioactivity) was used as the control. After 1, 3, 6, or 24 h of incubation, the cells were harvested and washed three times with cold PBS. Radioactivity was measured using a gamma counter. The cellular uptake radioactivity dose of ^89^Zr-Exo was calculated as follows: Percentage of added dose (%AD) = radioactivity of cell pellets/radioactivity of initial added dose × 100.

### 2.7. Animals

All animal experimental protocols were approved by the Institutional Animal Care and Use Committee (IACUC; No. BA-2101-311-002-01) of Seoul National University Bundang Hospital, Seongnam, Korea. Institute of cancer research (ICR) mice and Sprague–Dawley (SD) rats were purchased from Orient Bio (Seongnam, Korea). All animals were kept in a specific pathogen-free room maintained at ~21 °C, ~55% relative humidity, and a 12 h light/dark cycle, with food and water available ad libitum during the entire study period.

### 2.8. PET/CT Imaging of In Vivo-Administered ^89^Zr-Exo

A total of six ICR mice (males, *n* = 3, bodyweight (mean ± SD) = 35.6 ± 0.8 g; females, *n* = 3, body weight (mean ± SD) = 28.1 ± 1.4 g) and six SD rats (males, *n* = 3, bodyweight (mean ± SD) = 292.3 ± 21.3 g; females, *n* = 3, bodyweight (mean ± SD) = 247.9 ± 16.4 g) were used in the present study. A small-animal dedicated PET/CT system (NanoPET/CT, Mediso Inc., Budapest, Hungary) with a 10 cm-axial and 12 cm-transaxial field of view (FOV) was used. The PET spatial resolution was 1.2 mm full-width at half-maximum at the center of FOV. A CT scan (semi-circular full trajectory, maximum field of view, 480 projections, 50 kVp, 300 ms and 1:4 binning) was performed immediately before PET scan. The PET images were reconstructed using the iterative three-dimensional ordered subset expectation maximization algorithm and the following settings: 4 iterations, 6 subsets, full detector model, low regularization, spike filter on, voxel size 0.6 mm and 400–600 keV energy window. PET data were corrected for randoms, decay, scatter and attenuation during the reconstruction. Finally, the reconstructed PET and CT images had a volume of 142 mm × 142 mm × 163 mm and a voxel volume of 0.6 mm × 0.6 mm × 0.6 mm. Isoflurane (2% at 1 L/min oxygen flow) was used to induce and maintain anesthesia. The activity measured in each organ (becquerel, Bq) was normalized to the total injected activity to express the measured radioactivity as a percentage of the injected dose (%ID). A whole-body PET scan was performed immediately after administering an intravenous injection of ^89^Zr-Exo (ICR mice, 8.6 × 10^10^ particle number (pn)/kg (920.1 kBq) of ^89^Zr-Exo; SD rats, 7.5 × 10^10^ pn/kg (12117.4 kBq) of ^89^Zr-Exo). PET/CT images were acquired at 15 min, 1 h, 2 h, 6 h, 1 day, 2 days, 5 days, and 7 days after administration. The biodistribution and pharmacokinetics of ^89^Zr-Exo were evaluated in the major organs (the liver, spleen, brain, intestine, kidney, lung, stomach, urinary bladder, and heart) using a fused PET and CT image volume-of-interest (VOI) analysis. The radioactivity in each organ at each time point was extracted from the PET/CT imaging data using VOI-based analyses. The VOI analysis was performed using the PMOD software (ver 3.6, PMOD Technologies Ltd., Zurich, Switzerland). For the organs of interest, the pharmacokinetic parameters in each organ were quantitatively assessed: peak concentration (C_max_), time to reach C_max_ (T_max_), and area under the curve (AUC) of the time course of distribution.

### 2.9. Determining the Ex Vivo Biodistribution of ^89^Zr-Exo Using Gamma-Counting Assay

A total of 28 ICR mice (male, *n* = 14; female, *n* = 14) and 28 SD rats (male, *n* = 14; female, *n* = 14) were used in the present study. A gamma-counter system (1480 Wizard, PerkinElmer, MA, USA) was used for the radioactivity assays. Isoflurane (2% at 1 L/min oxygen flow) was used to induce and maintain anesthesia. Four ICR mice (two males and two females) and four SD rats (two males and two females) were sacrificed by carbon dioxide euthanasia at allocated time points (15 min, 1 h, 2 h, 6 h, 1 day, 2 days, and 7 days) after a single intravenous administration of ^89^Zr-Exo (ICR mice, fixed dose of 2.5 × 10^10^ pn/animal (1110 kBq) of ^89^Zr-Exo; SD rats, fixed-dose of 2.5 × 10^11^ pn/animal (11,100 kBq) of ^89^Zr-Exo). Various organs and tissues, as well as urine and blood, were harvested for subsequent gamma-counter assays. The activity measured in an organ (Bq) was normalized to the total injected activity to express the measured radioactivity as the percentage of the injected dose (%ID) or as the percentage of the injected dose per gram of tissue (%ID/g), with additional normalization to the tissue weight. The following pharmacokinetic parameters in each organ were quantitatively assessed for the organs of interest using the mean %ID or %ID/g value at the dedicated time point: peak concentration (C_max_), time to reach C_max_ (T_max_), and AUC of the time course of distribution.

### 2.10. Labeling Exosomes with ExoGlow Fluorescent Dye

Exosomes were labeled with the fluorescent dye ExoGlow^TM^-Vivo EV Labeling Kit (System Biosciences, Palo Alto, CA, USA) according to the manufacturer’s instructions. To remove the unlabeled free dye, ExoGlow-labeled exosomes were purified via size exclusion chromatography with Sepharose CL-6B resin. ExoGlow-labeled exosomes were obtained in fractions with labeled exosomes, and the free dye was consistently distinguished.

### 2.11. Imaging the In Vivo Biodistribution of Fluorescent Dye-Labeled Exosomes

Fluorescent dye-labeled exosomes were intravenously and intraperitoneally administered to 40 male C57BL/6 mice, which were sacrificed at 0.25, 1, 2, 24, and 72 h post-injection, and five organs (the liver, kidney, spleen, lung, and brain) were harvested in a time-dependent manner. The fluorescence intensity was measured using the VISQUE in vivo LF Smart (Vieworks, Anyang, Korea) in vivo imaging equipment. The intensity unit of the organ image was expressed as a reliable intensity value by calculating the radiant efficiency (RE). Distribution analysis was performed using the sum value of the RE (not the average value) by unifying the area of the organ of all animals. The distribution value of all organs was normalized to the autofluorescence of normal organs.

## 3. Results

### 3.1. Characterization of GMP-Grade Therapeutic Exosomes Radiolabeled with ^89^Zr

**Figure 1:**
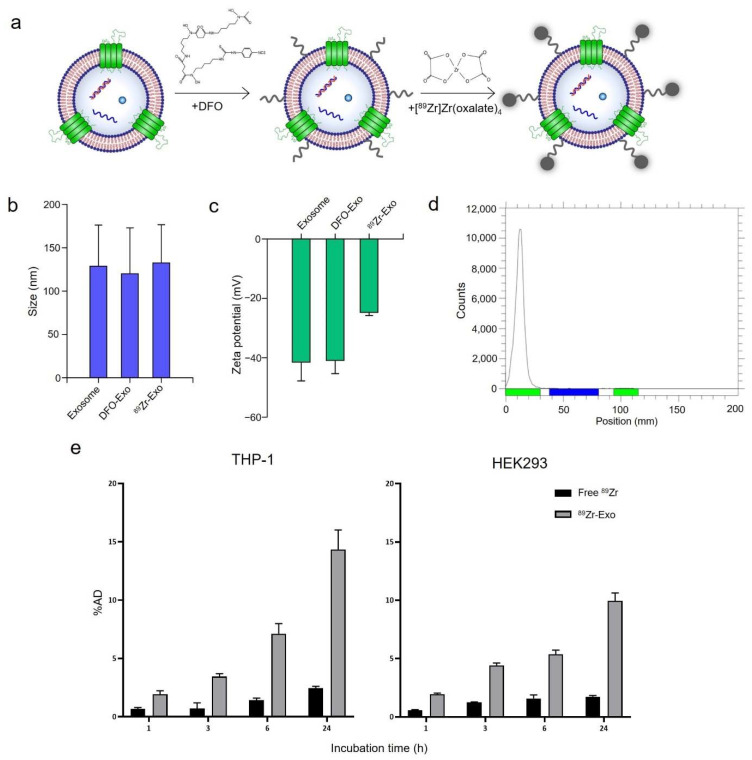
Radiolabeling GMP-grade exosomes with ^89^Zr. (**a**) Schematic of the method for labeling the surface of exosomes with ^89^Zr. (**b**) ^89^Zr-Exo size was measured using NTA shown as mean ± standard deviation (SD). (**c**) Zeta potential of ^89^Zr-Exo was measured using Zetasizer Nano ZS shown as mean ± SD. (**d**) Radiochemical purity of ^89^Zr-Exo was analyzed using radio-TLC. (**e**) In vitro cellular uptake of ^89^Zr-Exo was evaluated in the THP-1 and HEK293 cell lines at the indicated time points shown as %AD ± SD.

To produce GMP-grade therapeutic exosomes with high purity and minimal batch-to-batch variance, exosomes were isolated using a combination of filtration, anion exchange chromatography, and size-exclusion chromatography (Figure S1a). Isolated exosomes contained exosome markers, such as CD9, CD81, Alix, and TSG101, whereas negative markers, such as GM130 and Lamin B1, were not detected (Figure S1b,c). Monitoring batch-to-batch variances indicated that the exosomes isolated from different batches were of consistent quality (Table S1). The quality control criteria, such as particle number, protein amount, and purity were determined based on general factors that are used to characterize exosomes and biologics [6]. Surface labeling of ^89^Zr was conducted by first conjugating the exosomes with a DFO chelator, which binds to the lysine residues of the transmembrane proteins of exosomes via its amine-reactive groups. The exosomes were then radiolabeled with ^89^Zr. ^89^Zr-Exo was similar in size (128.8 ± 47.4 nm) to non-labeled exosomes (132.6 ± 44.2 nm, Figure 1b). In addition, the zeta potential of ^89^Zr-Exo was similar to that of the non-labeled exosomes (Figure 1c), thus confirming that no significant change in physicochemical properties occurred during the radiolabeling process. Radio-TLC analysis showed that the radiochemical purities of ^89^Zr-Exo were 93.6 ± 1.1% (mouse batch) and 97.7 ± 2.2% (rat batch) (Figure 1d). In addition, ^89^Zr-Exo demonstrated potent cellular uptake after surface labeling of radioisotopes, showing 14% uptake in the THP-1 human monocytic leukemia cell line and 10% uptake in the HEK293 human embryonic kidney cell line at 24 h (Figure 1e).

### 3.2. Biodistribution and PK of ^89^Zr-Exo in Mouse Using PET/CT Imaging

**Figure 2:**
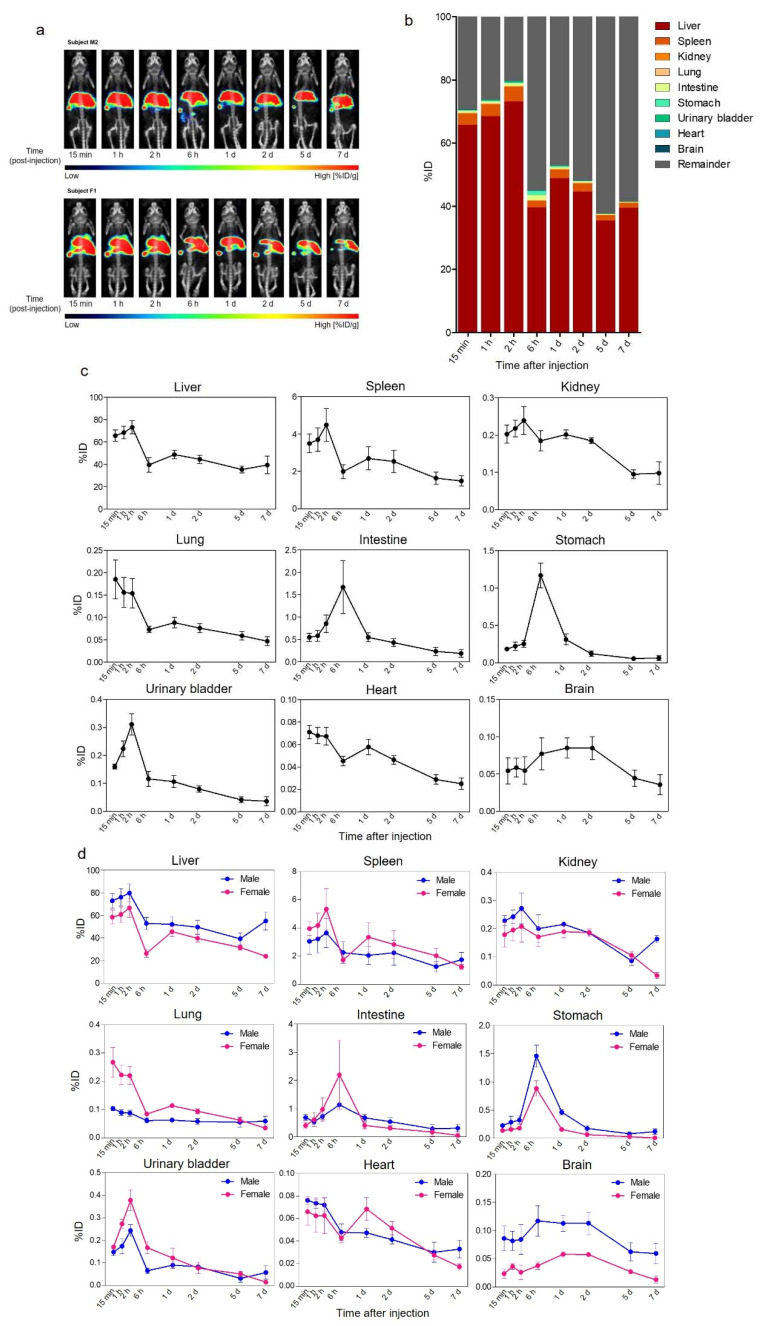
Biodistribution of ^89^Zr-labeled GMP-grade exosomes in mice imaged using PET/CT. (**a**) Biodistribution of i.v.-administered ^89^Zr-Exo in mice was evaluated using PET/CT imaging, and the values are shown as the %ID/g at various time points. (**b**) Whole-body fraction of ^89^Zr-Exo in various organs of mice (*n* = 6) was determined by a volume-of-interest analysis of the PET/CT image, and the values are shown as the %ID at various time points. (**c**) Time-course distribution of ^89^Zr-Exo was shown in each organ as the %ID with standard error of mean (SEM). (**d**) Time-course distribution of ^89^Zr-Exo in male (*n* = 3) and female (*n* = 3) mice was shown in each organ as the %ID with SEM.

We analyzed the in vivo biodistribution and PK of ^89^Zr-Exo in mice using PET coupled with CT imaging. Six ICR mice underwent whole-body PET/CT at multiple time points (15 min, 1 h, 2 h, 6 h, 1 day, 2 days, 5 days, and 7 days) after a single intravenous (i.v.) injection of ^89^Zr-Exo. ^89^Zr-Exo showed a prompt and predominant distribution in the liver and spleen after systemic administration, and this distribution was maintained for up to 7 days with only minor clearance of ^89^Zr (Figure 2a). Prolonged accumulation of ^89^Zr was not observed in mice injected with free ^89^Zr conjugated with DFO only (^89^Zr-DFO), which was rapidly excreted through the urinary bladder, stomach, gallbladder, and intestine, and the ^89^Zr signal was almost undetectable at 2 days after administration (Figure S2). Quantitative pharmacokinetic analysis of visually identified organs using PET and CT images showed a high accumulation of ^89^Zr-Exo in the liver and spleen, with a peak intensity of approximately 73% of the injected dose (ID) and 5%ID, respectively, reached within 2 h after administration (Figure 2b,c, Table 1). The predominant liver accumulation was also observed in both i.v. and intraperitoneally (i.p.) administered ExoGlow lipophilic fluorescent dye-labeled exosomes; however, i.v. injection led to a higher accumulation in the liver than i.p. injection (Figure S3). In addition, less than one-third of the total fluorescence intensity was observed at 72 h after administration compared with the total intensity at 15 min, indicating a loss of fluorescence signal over time (Figure S3). The intestine and stomach showed a high accumulation of ^89^Zr-Exo, with a peak intensity observed at approximately 6 h. Moderate accumulation was observed in the kidney, lung, and heart. A marginally 6-fold greater AUC was observed in the intestine (including feces) than in the urinary bladder (including urine). Approximately 0.1% of ^89^Zr-Exo was delivered to the brain, which has been reported in other studies [22,62,63]. Comparative analysis of the biodistribution between different gender showed that male mice showed increased accumulation of exosomes in the liver (*p* < 0.01), kidney (*p* < 0.05), stomach (*p* < 0.05), and brain (*p* < 0.0001), whereas female mice showed increased accumulation in the lungs (*p* < 0.05, Figure 2d).

### 3.3. Biodistribution and PK of ^89^Zr-Exo in Mouse Using Ex Vivo Gamma-Counting Analysis

**Figure 3:**
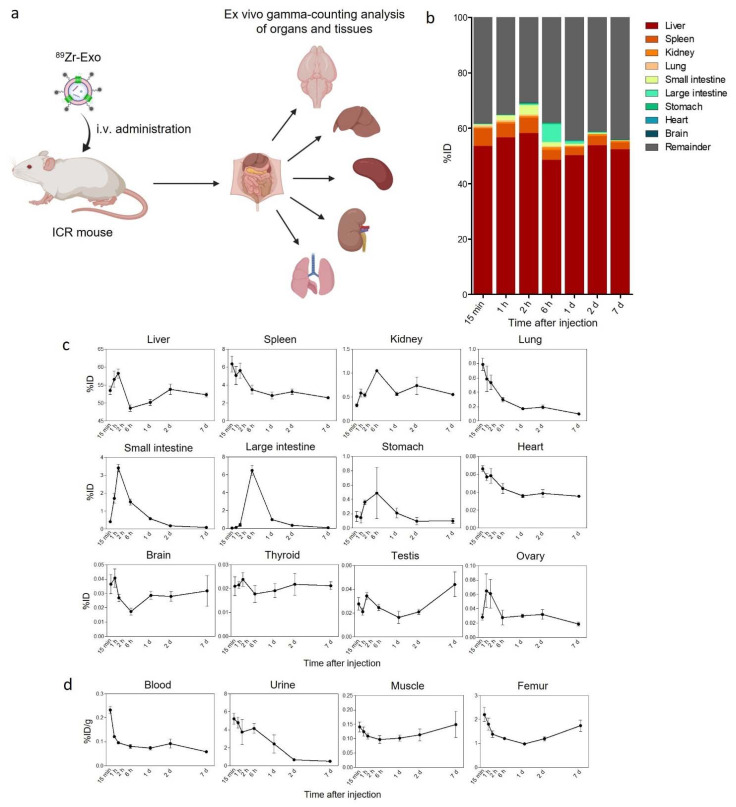
Biodistribution of ^89^Zr-labeled GMP-grade exosomes in mice analyzed using ex vivo gamma-counting analysis. (**a**) Schematic of the ex vivo gamma-counting analysis of the distribution of i.v.-administered ^89^Zr-Exo in various organs and tissues of mice. (**b**) Whole-body fraction of ^89^Zr-Exo in various organs of mice (*n* = 4 for each time point) was determined using a gamma-counting assay, with the results shown as the %ID at various time points. (**c**) Time-course distribution of ^89^Zr-Exo in each organ determined using a gamma-counting assay was shown as the %ID with SEM. (**d**) Time-course distribution of ^89^Zr-Exo in the blood, urine, muscle, and femur was shown as the %ID/g with SEM due to differences in the sampling methods.

We performed an ex vivo gamma-counting analysis to determine the biodistribution and PK of ^89^Zr-Exo in mice to elucidate the quantitative and detailed pharmacokinetic properties in the organs and tissues of interest that could not be revealed using PET/CT imaging. Following i.v. administration of ^89^Zr-Exo, four animals were sacrificed at allocated time points, and various organs, tissues, urine, and blood were collected for the subsequent gamma-counting assay. The results demonstrated similar organ distribution patterns as the PET/CT imaging data and illustrated that the liver and spleen were the predominant organs showing exosome accumulation (Figure 3a–c, Table 2). The small intestine showed peak intensity at 2 h, whereas the large intestine showed peak intensity at 6 h, which was probably because of the movement of exosome-containing feces from the small intestine to the large intestine. A moderate distribution of ^89^Zr-Exo was observed in the kidneys, lungs, stomach, brain, and heart. ^89^Zr-Exo also accumulates in the thyroid, testes, and ovaries. Attention should be paid to the fact that different units (%ID/g, not %ID) were used to indicate the level of distribution in the blood, urine, muscle, and femur because of differences in the sampling methods (Figure 2d). ^89^Zr-Exo was rapidly cleared from the blood circulation, which was consistent with other studies analyzing the blood PK of exosomes [24,27,37,55]. A significant amount of ^89^Zr-Exo was observed in the urine, which showed a peak at 15 min and rapid decreases over time.

### 3.4. Biodistribution and PK of ^89^Zr-Exo in Rats Using PET/CT Imaging

**Figure 4:**
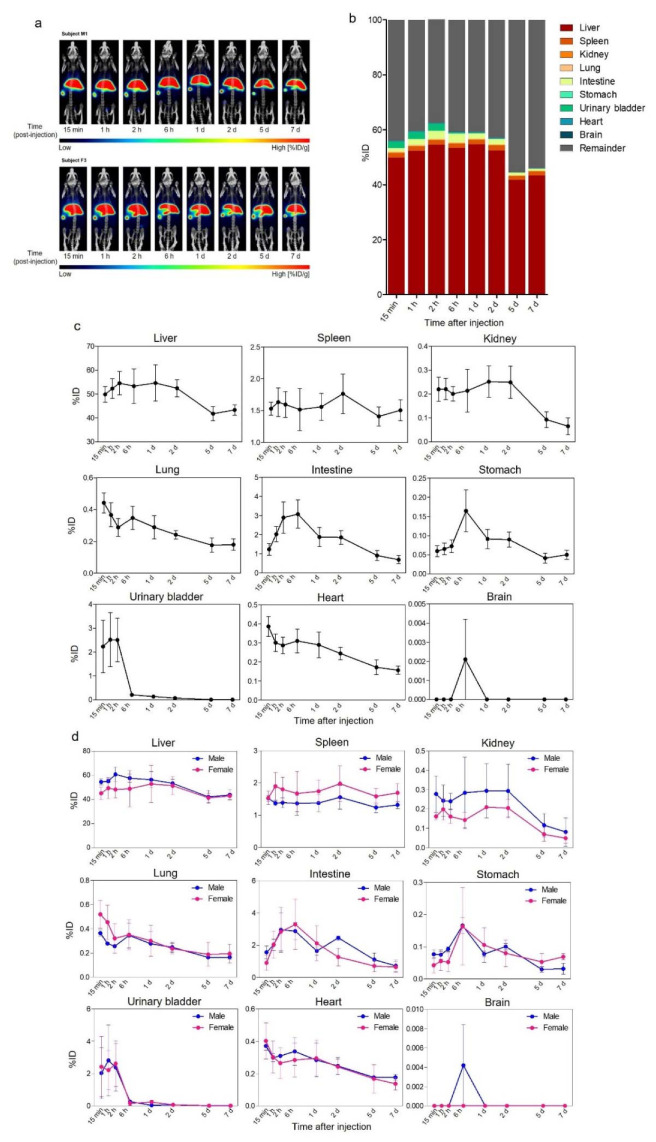
Biodistribution of ^89^Zr-labeled GMP-grade exosomes in rats imaged by PET/CT. (**a**) Biodistribution of i.v.-administered ^89^Zr-Exo in rat was evaluated at various time points through PET/CT imaging, with the results shown as the %ID/g. (**b**) Whole-body fraction of ^89^Zr-Exo in various organs of rat (*n* = 6) was assessed by a volume-of-interest analysis of the PET/CT image, with the results shown as the %ID at various time points. (**c**) Time-course distribution of ^89^Zr-Exo in each organ was shown as the %ID with SEM. (**d**) Time-course distribution of ^89^Zr-Exo in male (*n* = 3) and female (*n* = 3) rats was shown in each organ as the %ID with SEM.

Six SD rats underwent whole-body PET/CT scans at multiple time points after i.v. administration of ^89^Zr-Exo (Figure 4a–c, Table 3). Consistent with the biodistribution in mice, ^89^Zr-Exo was predominantly distributed in the liver and spleen in the rats; however, the peak intensity was lower than that in mice (64%ID vs. 73%ID and 2%ID vs. 5%ID, respectively). An increased distribution in the urinary bladder and intestine was observed in rats compared to that in mice (2.95%ID vs. 0.31%ID and 3.71%ID vs. 1.81%ID, respectively), which implied increased urinary or intestinal excretion of ^89^Zr-Exo in rats. A moderate accumulation was observed in the kidneys, lungs, and heart. Almost no accumulation of ^89^Zr-Exo was observed in the rat brains. Similar with the case of male mice, male rats demonstrated increased accumulation of exosomes in the liver (*p* < 0.001) and kidney (*p* < 0.01) compared to female rats (Figure 4d). Female rats showed increased accumulation in the spleen (*p* < 0.001) and lungs (*p* < 0.05) (Figure 4d).

### 3.5. Biodistribution and PK of ^89^Zr-Exo in Rats Using Ex Vivo Gamma-Counting Analysis

**Figure 5:**
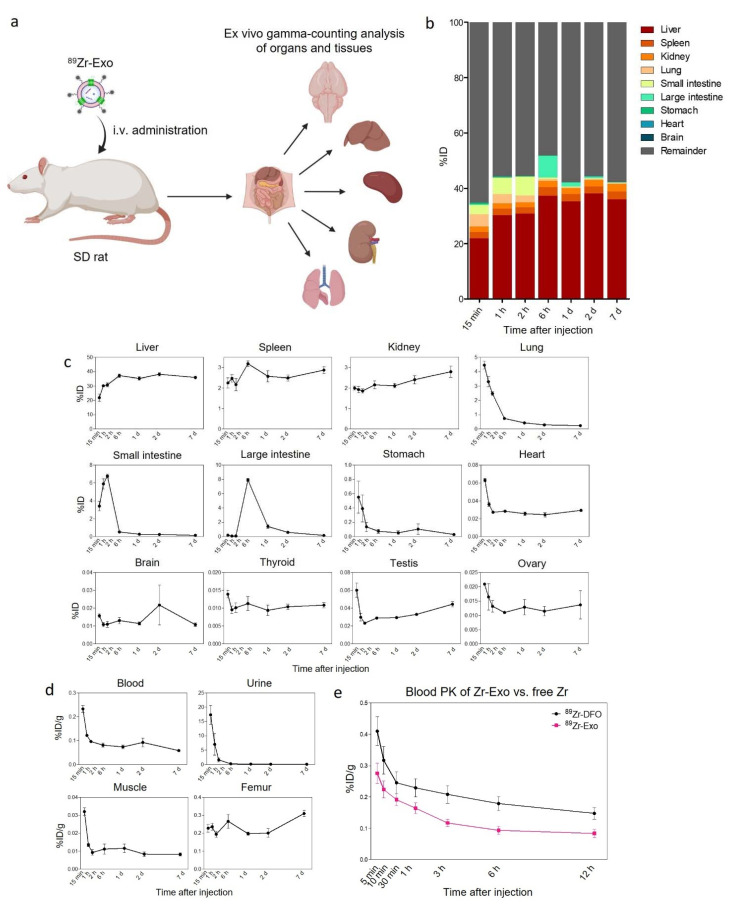
Biodistribution and blood PK of ^89^Zr-labeled GMP-grade exosomes in rats based on ex vivo gamma-counting analysis. (**a**) Schematic of the ex vivo gamma-counting analysis of the distribution of i.v.-administered ^89^Zr-Exo in various organs and tissues of rats. (**b**) Whole-body fraction of ^89^Zr-Exo in various organs of rats (*n* = 4 for each time point) was determined using a gamma-counting assay, with the results shown as the %ID at various time points. (**c**) Time-course distribution of ^89^Zr-Exo obtained using a gamma-counting assay in each organ was shown as the %ID with SEM. (**d**) Time-course distribution of ^89^Zr-Exo in the blood, urine, muscle, and femur was shown as the %ID/g with SEM due to differences in the sampling methods. (e) Time-course distribution of ^89^Zr-Exo and free ^89^Zr-DFO in the blood was shown as the %ID with SEM.

We analyzed the biodistribution and PK of ^89^Zr-Exo in rats based on an ex vivo gamma counting analysis. Following i.v. administration of ^89^Zr-Exo, four animals were sacrificed at allocated time points, and then various organs, tissues, urine, and blood were collected for the subsequent gamma-counting assay (Figure 5a–d, Table 4). Consistent with the PET/CT imaging data, the liver was the predominant organ that exhibited the highest exosome accumulation. However, the peak intensity of the liver was lower than that based on the PET/CT data (38%ID vs. 64%ID). In comparison, an increased peak intensity in the rat lungs and kidneys was observed using gamma counting compared with PET/CT imaging (4.45%ID vs. 0.49%ID; 2.79%ID vs. 0.34%ID, respectively). Consistent with the case in mice, the peak intensities of the small and large intestine were reached at 2 h and 6 h, respectively. However, the distributions of ^89^Zr-Exo in the kidney and urine were increased in rats compared to that in mice, with a peak intensity of 2.79%ID vs. 1.05%ID for the kidney and 17.33%ID/g vs. 1.05%ID/g for the urine. Also, rapid clearance of ^89^Zr-Exo from the blood circulation was observed in rats, with an AUC of 0.09 for rats vs. 0.53 for mice. To quantitatively analyze the blood PK of ^89^Zr-Exo compared with that of free ^89^Zr, the early time-course blood PK of i.v.-administered ^89^Zr-Exo and ^89^Zr-DFO was determined in rats (Figure 5e). Substantial differences in the blood PK were observed between ^89^Zr-Exo and ^89^Zr-DFO, with ^89^Zr-Exo demonstrating rapid clearance from whole blood within 5 min.

## 4. Discussion

We assessed the preclinical biodistribution and PK of GMP-grade therapeutic exosomes by radiolabeling their surface with ^89^Zr. The characteristics of exosomes are largely affected by the isolation methods because of the overlapping physical properties of EVs [52]. In addition, careful consideration is warranted for rigorous monitoring of culture conditions for exosome-producing cells because minor changes in the conditions of parental cells will also affect the content of exosomes produced [53,54]. For the first time, we evaluated the biodistribution of GMP-grade exosomes isolated using a combination of filtration, anion exchange chromatography, and size-exclusion chromatography. Although most biodistribution studies using radionuclide imaging have assessed the distribution in mice, we observed the quantitative biodistribution of radiolabeled exosomes not only in mice but also in rats. Exosomes are novel nanoparticles that require intensive evaluation not only to determine their therapeutic efficacy but also to reveal their safety and bioavailability. Quantitative monitoring of the in vivo fate of exosomes in various animal models through radionuclide imaging will accelerate the clinical translation of therapeutic exosomes.

Although several previous studies radiolabeled exosomes using the intraluminal method to conduct biodistribution analyses [27,51], we observed that the surface labeling method presents superior stability when exosomes are radiolabeled with ^89^Zr compared with the intraluminal labeling method (data not shown). Whereas several previous studies used %ID per gram of tissue for analyzing the biodistribution of exosomes, we used %ID of the whole tissue to quantitatively compare the accumulation of exosomes between tissues. The quantitative biodistribution analysis of ^89^Zr-Exo administered to rodents (ICR mice and SD rats) revealed that exosomes are mainly distributed in the liver and, to a lesser extent, the spleen, which was consistent with the findings of previous studies [22,23,24,25,26,27,28,29,30]. A moderate distribution of ^89^Zr-Exo was observed in the kidneys, lungs, stomach, brain, and heart. Although several studies showed accumulation of exosomes in the lungs of mice [47,49], we found minimal accumulation of exosomes in the lungs. This was consistent with our previous study which showed that significant amount of exosomes were accumulated in the lungs of mice only under septic conditions [23]. ^89^Zr-Exo also accumulated in the thyroid, testes, and ovaries, which was newly observed in our study. The small intestine exhibited a peak intensity at 2 h, while the large intestine showed a peak intensity at 6 h, which was probably due to the movement of exosome-containing feces. Consistent biodistribution patterns of ^89^Zr-Exo were observed in mice based on PET/CT imaging and gamma counting. Rats showed discrete patterns in organs such as the liver, kidney, and lung based on the PET/CT imaging and gamma-counting analyses. This discrepancy could occur due to loss of sensitivity of gamma counter with increasing sample volume [64]. When analyzing blood samples, this effect can be minimized by matching the sample volume [65]. However, this approach may not be feasible when analyzing solid tissue samples. which urge caution when quantitatively interpreting radionuclide activity in organs of rats.

Generally, mice and rats demonstrated similar time-course biodistribution patterns but the total ^89^Zr signal in the organs was lower in rats than in mice, suggesting a higher excretion rate of ^89^Zr-Exo in rats. Mice showed a higher distribution in the liver than rats, with a peak liver intensity of 58%ID for mice and 38%ID for rats based on the gamma-counting analysis (*p* < 0.001). A similar tendency was observed for the spleen, which exhibited a peak intensity of 6%ID for mice and 3%ID for rats (*p* = 0.052). In addition, ^89^Zr-Exo showed increased urinary excretion in rats as shown by the increased accumulation in the kidney and urine compared to that in mice. The difference in the organ weight-to-body weight ratio could also be the reason for lower exosome accumulation in the organ of rats, as liver weights typically fall in the 3–5% body weight in mice, and 2–3% body weight in rats, which could result in lower %ID in the liver of rats compared to that of mice.

A prolonged ^89^Zr signal for up to 7 days was observed in most organs, such as the liver, spleen, kidney, and heart, which indicated that most of the exosomes were taken up by cells in those organs, thus underscoring the therapeutic potential of exosomes for intracellular delivery of therapeutics. In contrast, a rapid decline in the ^89^Zr-Exo signal was observed in other organs, such as the lung, intestine, stomach, and urinary bladder, implying that ^89^Zr-Exo bypasses these organs and is not taken up by the cells or proceeds directly to excretion. The reason for the prolonged ^89^Zr signal observed after ^89^Zr-Exo administration requires further investigation. Very low levels of ^89^Zr-Exo accumulation were observed in the brain, thyroid, testes, and ovaries, indicating that additional targeting modifications are required for the delivery of exosomes to these organs. ^89^Zr-Exo demonstrated rapid clearance from the whole blood within minutes, which was consistent with previous reports [24,27,37,55]. Notably, ^89^Zr presented bone-seeking behavior due to its strong affinity for phosphate, and this behavior led to an increased distribution of free ^89^Zr in the femur (Figure S2). ^89^Zr-Exo was cleared from the circulation more rapidly than free ^89^Zr, which indicates that ^89^Zr-Exo was rapidly taken up into cells and tissues while ^89^Zr-DFO was mostly destined for urinary and biliary excretion (Figure S2).

Recent studies have identified molecules that constitute the membrane of exosomes which determine their cellular or organ tropisms [66,67]. The major proteins that are displayed on exosomal membranes are tetraspanins (e.g., CD9, CD63, CD81, and CD82) and integrins, of which various compositions of these proteins could influence the organotropism of exosomes [7]. In addition, the pathophysiological conditions of the host can affect the biodistribution and PK parameters of exosomes. We previously found that a substantial number of exosomes were delivered to the lungs of sepsis-induced mice while very low levels were detected in the lungs of healthy mice [23]. However, most studies evaluating the biodistribution of exosomes used fluorescence or bioluminescence imaging methods to measure cellular or organ distribution. Tracking the in vivo fate of exosomes using radionuclide imaging will allow for a quantitative analysis of the biodistribution and PK, which will help decode the molecular mechanisms underlying the cellular and organ tropism of exosomes.

Recent advances have highlighted the potential of targeted delivery of exosomes to various organs, including the central nervous system, which is one of the most challenging organs for drug delivery [31,63]. To induce targeted delivery, the surface of exosomes can be engineered to express various targeting moieties via direct chemical modification or indirect modification by genetically modifying exosome-producing cells [68]. Radionuclide imaging will allow for quantitative evaluations of the effect of surface modifications on the targeted delivery of exosomes. However, caution must be exercised when labeling radioisotopes on exosomes expressing targeting moieties because such labeling might affect the targetability because of interactions with targeting moieties.

## Figures and Tables

**Table 1:** In vivo pharmacokinetic parameters of ^89^Zr-Exo following i.v. administration in various organs of ICR mice.

Organ	C_max_ [%ID] (*n*)	T_max_ [d] (*n*)	AUC_0-t_ [%ID × d] (*n*)
Liver	73.19 ± 14.27 (6)	0.08 ± 0.00 (6)	288.69 ± 70.48 (6)
Spleen	4.64 ± 2.03 (6)	0.08 ± 0.02 (6)	14.37 ± 6.83 (6)
Brain	0.09 ± 0.04 (6)	1.04 ± 0.56 (6)	0.42 ± 0.22 (6)
Intestine	1.81 ± 1.40 (6)	0.22 ± 0.07 (6)	2.73 ± 1.25 (6)
Stomach	1.17 ± 0.40 (6)	0.25 ± 0.00 (6)	1.17 ± 0.59 (6)
Kidney	0.26 ± 0.07 (6)	0.42 ± 0.78 (6)	0.98 ± 0.12 (6)
UB	0.31 ± 0.09 (6)	0.08 ± 0.00 (6)	0.45 ± 0.20 (6)
Lung	0.19 ± 0.11 (6)	0.02 ± 0.03 (6)	0.48 ± 0.13 (6)
Heart	0.08 ± 0.01 (6)	0.20 ± 0.39 (6)	0.27 ± 0.04 (6)

Values are the mean ± SD.

**Table 2:** Ex vivo pharmacokinetic parameters of ^89^Zr-Exo following i.v. administration in various organs of ICR mice.

Organ	C_max_ [%ID]	T_max_ [d]	AUC_0–t_ [%ID × d]
Liver	58.22	0.08	367.59
Spleen	6.35	0.01	21.13
Brain	0.04	0.04	0.20
Small intestine	3.41	0.08	2.23
Large intestine	6.49	0.25	4.19
Stomach	0.49	0.25	0.96
Kidney	1.05	0.25	4.63
Urine *	*5.22*	*0.01*	*7.65*
Lung	0.79	0.01	1.17
Heart	0.07	0.01	0.27
Thyroid	0.02	0.08	0.15
Testis	0.04	7.00	0.20
Ovary	0.06	0.04	0.19
Muscle *	*0.15*	*7.00*	*0.86*
Femur *	*2.20*	*0.01*	*9.59*
Blood *	*0.23*	*0.01*	*0.53*

* Unit (concentration) = %ID/g.

**Table 3:** In vivo pharmacokinetic parameters of ^89^Zr-Exo following i.v. administration in various organs of SD rats.

Organ	C_max_ [%ID] (*n*)	T_max_ [d] (*n*)	AUC_0-t_ [%ID × d] (*n*)
Liver	63.58 ± 12.67 (6)	1.56 ± 2.70 (6)	332.14 ± 50.00 (6)
Spleen	2.00 ± 0.65 (6)	3.02 ± 3.21 (6)	10.82 ± 3.15 (6)
Brain	0.00 ± 0.01 (6)	0.25 ± NA (1)	0.00 ± 0.00 (6)
Intestine	3.71 ± 1.84 (6)	0.61 ± 0.76 (6)	9.58 ± 4.67 (6)
Stomach	0.18 ± 0.12 (6)	1.50 ± 2.71 (6)	0.48 ± 0.28 (6)
Kidney	0.34 ± 0.17 (6)	0.60 ± 0.77 (6)	1.03 ± 0.84 (6)
UB	2.95 ± 2.91 (6)	0.06 ± 0.03 (6)	0.60 ± 0.32 (6)
Lung	0.49 ± 0.16 (6)	0.22 ± 0.39 (6)	1.58 ± 0.61 (6)
Heart	0.42 ± 0.13 (6)	0.22 ± 0.40 (6)	1.55 ± 0.53 (6)

Values are the mean ± SD.

**Table 4:** Ex vivo pharmacokinetic parameters of ^89^Zr-Exo following i.v. administration in various organs of SD rats.

Organ	C_max_ [%ID]	T_max_ [d]	AUC_0-t_ [%ID × d]
Liver	38.13	2.00	256.82
Spleen	3.19	0.25	18.73
Brain	0.02	2.00	0.11
Small intestine	6.77	0.08	2.22
Large intestine	7.91	0.25	6.04
Stomach	0.55	0.01	0.46
Kidney	2.79	7.00	17.37
Urine *	*17.33*	*0.01*	*1.46*
Lung	4.45	0.01	2.57
Heart	0.06	0.01	0.19
Thyroid	0.01	0.01	0.07
Testis	0.06	0.01	0.25
Ovary	0.02	0.01	0.09
Muscle *	*0.03*	*0.01*	*0.06*
Femur *	*0.31*	*7.00*	*1.70*
Blood *	*0.12*	*0.01*	*0.09*

* Unit (concentration) = %ID/g.

## Data Availability

The datasets generated and/or analyzed during the current study are available from the corresponding author upon reasonable request.

## Supplementary Materials

The following supporting information can be downloaded at: https://www.mdpi.com/article/10.3390/pharmaceutics14061118/s1, Figure S1: Production and characterization of therapeutic exosomes; Figure S2: Biodistribution of free ^89^Zr; Figure S3: Biodistribution of fluorescent dye-labeled GMP-grade exosomes; Table S1: Criteria for confirming batch-to-batch variances of therapeutic exosomes.
