# Supplementary material for: Quantitative Biodistribution and Pharmacokinetics Study of GMP-Grade Exosomes Labeled with 89Zr Radioisotope in Mice and Rats
Source: Pharmaceutics. 2022 May 24;14(6):1118. doi: 10.3390/pharmaceutics14061118 (PMC9229812; doi:10.3390/pharmaceutics14061118)
Supplement: Supplementary file 1 [file pharmaceutics-14-01118-s001.zip › pharmaceutics-1701709-supplementary.pdf]

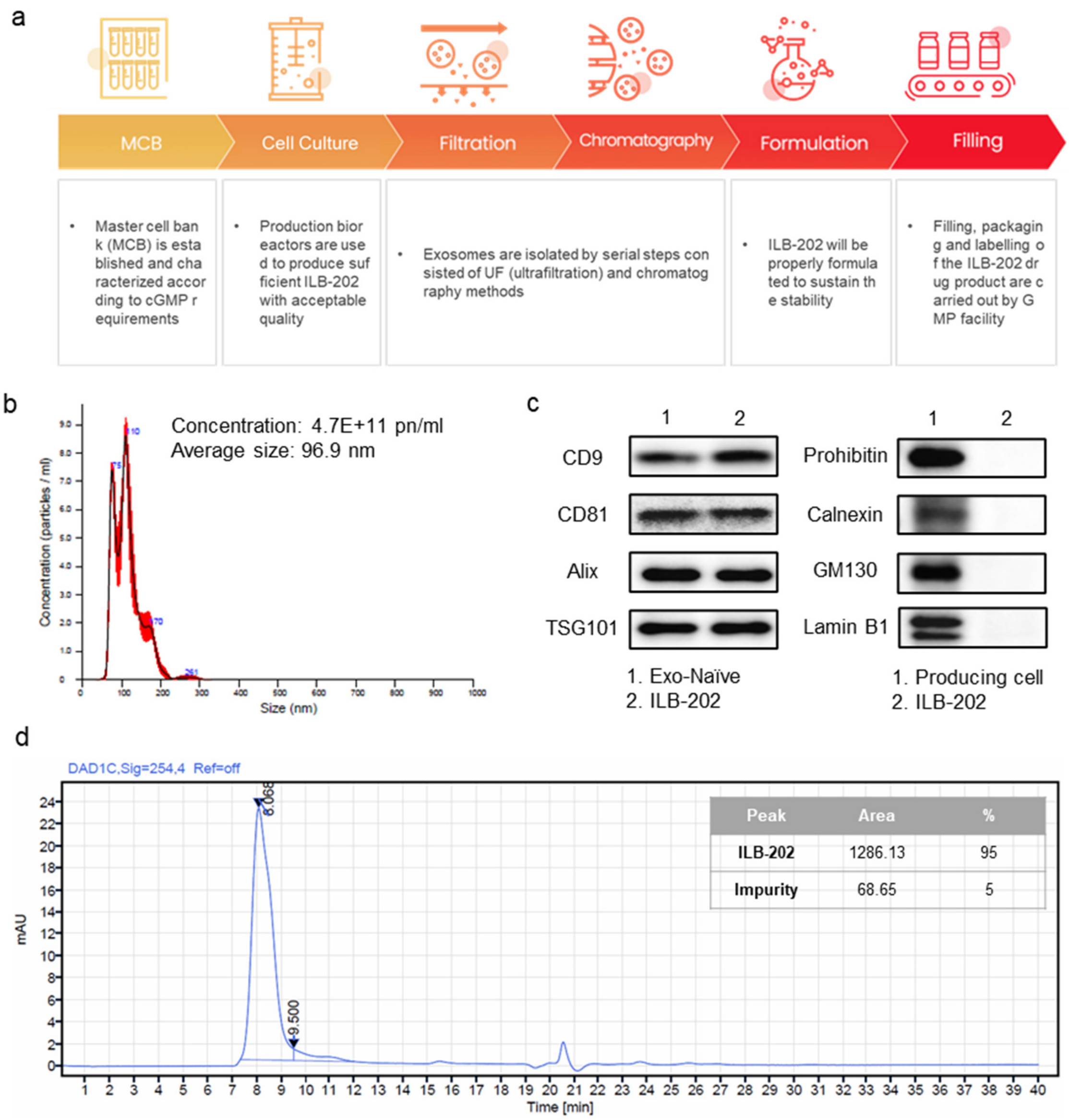

Figure S1. Production and characterization of therapeutic exosomes. (**a**) Schematics of isolation process for therapeutic exosomes are shown. (**b**) Size of ILB-202 were measured by NTA. (**c**) Positive and negative markers for exosomes were analyzed in naturally produced exosomes (Exo-Naïve) and ILB-202 by immunoblotting. (**d**) Purity of ILB-202 were analyzed by high performance liquid chromatography.

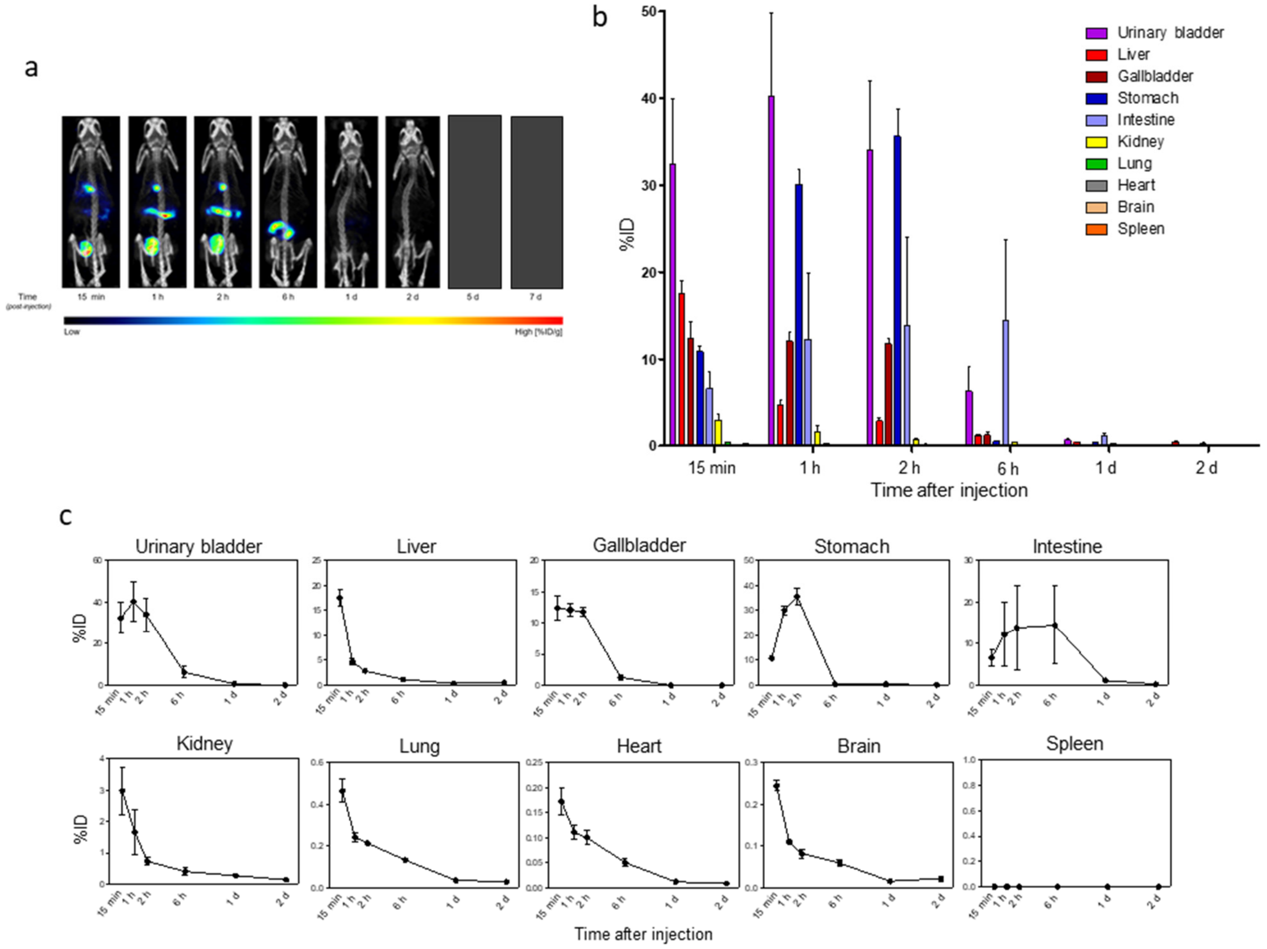

Figure S2. Biodistribution of free ^{89}Zr. (**a**) Biodistribution of i.v. administrated ^{89}Zr-DFO in mice were evaluated by %ID/g at various time points through PET/CT imaging. (**b**) Time-course distribution of ^{89}Zr-DFO in various organs of mice were analyzed by volume-of-interest analysis extracted from the PET/CT image, which are shown as %ID with SEM at various time points. (**c**) Time-course distribution of ^{89}Zr-DFO were shown in each organs as %ID with SEM.

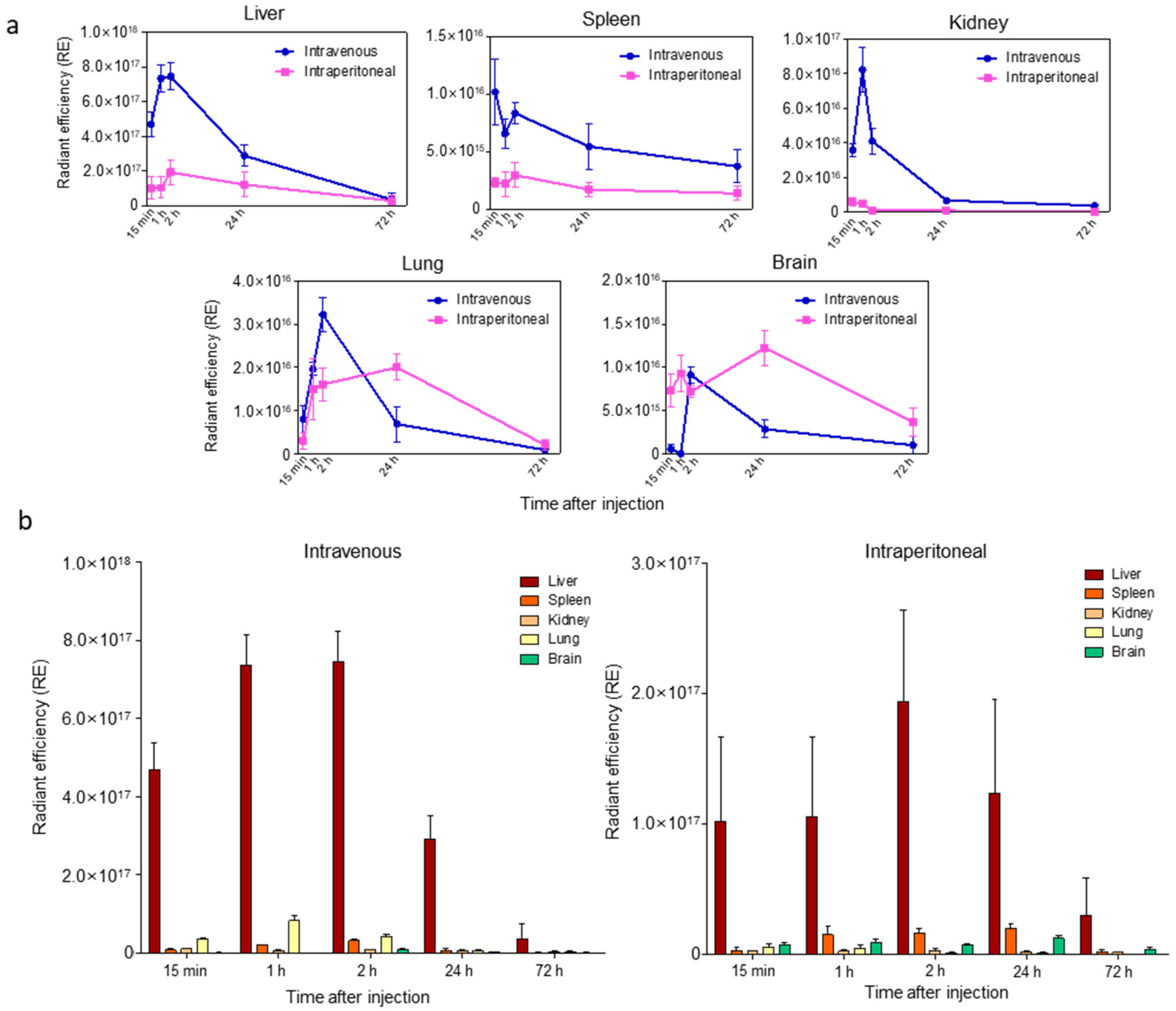

Figure S3. Biodistribution of fluorescent dye-labeled GMP-grade exosomes (**a**) Time-course biodistribution ExoGlow-labeled exosomes administered by i.v. or i.p. route in mice were evaluated by measuring radiant efficiency at various time points through in vivo fluorescence imaging (mean ± SEM). (**b**) Time-course distribution of ExoGlow-labeled exosomes were organized by injection route (mean ± SEM).

Table S1. Criteria for confirming batch-to-batch variances of therapeutic exosomes.

Summary	Tests	Acceptance Criteria	Batch A	Batch B
**General properties**	Appearance	Colorless to yellow and clear to opalescent solution that may contain translucent to white particles	Pass	Pass
	pH	7.5 to 8.5	7.8	7.8
**Identity**	srIκB protein	Pass	Pass	Pass
**Strength**	Exosome particle number	4.0E+11 to 6.0E+11 pn/mL	4.7E+11	4.3E+11
	Total protein	Report result	138.4 μg/mL	119.6 μg/mL
	srIκB protein concentration	Report result	1.0 μg/mL	1.0 μg/mL
**Purity**	Purity	≥ 90 %	95 %	96 %
**Potency**	In vitro TNF-α assay	50 < relative potency (%) < 150	94 %	110 %
**Safety**	Endotoxin	≤ 5 EU/mL	< 0.5 EU/mL	< 5 EU/mL
	Sterility	No growth	No growth	No growth
